# The association between time-weighted cumulative life's essential 8 and aortic valve calcification

**DOI:** 10.3389/fcvm.2026.1763288

**Published:** 2026-05-29

**Authors:** Man Gui, Shouling Wu, Tonghaoyu Wang, Yihao Qi, Zhenyu Huo, Ying Gao, Haiyan Zhang, Ying Han, Jialing Deng, Jing Dong, Taiying Wang, Shuohua Chen, Fengmei Xing

**Affiliations:** 1 School of Nursing and Rehabilitation, North China University of Science and Technology, Tangshan, Hebei, China; 2 Department of Cardiology, Kailuan General Hospital, Tangshan, Hebei, China; 3 Medical Imaging Department, Children’s Hospital Affiliated to Shandong University, Jinan, China; 4 School of Public Health, North China University of Science and Technology, Tangshan, China; 5 Department of Nursing, North China University of Science and Technology Affiliated Hospital, Tangshan, Hebei, China; 6 School of Clinical, North China University of Science and Technology, Tangshan, Hebei, China; 7 Department of Spine and Spinal Cord, Affiliated Hospital of North China University of Science and Technology, Tangshan, Hebei, China; 8 Shandong Public Health Clinical Center, Shandong University, Jinan, China

**Keywords:** aortic valve calcification, cardiovascular health, Kailuan study, LE8, time-weighted cumulative Life's Essential 8 scores

## Abstract

**Objective:**

To examine the association between the time-weighted cumulative Life's Essential 8 (TWC-LE8) score and aortic valve calcification (AVC).

**Methods:**

This analysis included 15,321 adults (mean age, 63.79 years; 76.44% men) from the Kailuan cohort who completed four health examinations between 2006 and 2012 and subsequently underwent standardized echocardiography. The TWC-LE8 score (range, 0–100) was used to classify cardiovascular health (CVH) as low (<50), moderate (50–79), or high (≥80). Multivariable logistic regression was used to estimate odds ratios (ORs) for AVC across CVH categories, and restricted cubic spline models were applied to assess the dose–response relationship.

**Results:**

A total of 2,894 AVC cases were identified. Compared with low CVH, moderate CVH was associated with lower odds of AVC (OR = 0.53, 95% CI: 0.47–0.61), and high CVH was associated with even lower odds of AVC (OR = 0.25, 95% CI: 0.15–0.40). A linear inverse association between TWC-LE8 score and AVC was observed (P for trend < 0.001). Age-stratified analyses showed significant heterogeneity. Among participants aged ≥55 years, high CVH was associated with 74% lower odds of AVC (OR = 0.26, 95% CI: 0.15–0.45), whereas no significant association was observed among those aged <55 years.

**Conclusion:**

Higher cumulative LE8 scores were strongly associated with lower odds of AVC, particularly among older adults. These findings support an inverse association between long-term cardiovascular health and AVC.

## 1 Introduction

With the accelerating aging of the global population, aortic valve calcification (AVC) has become an increasingly important contributor to the burden of cardiovascular disease. According to the Global Burden of Disease (GBD) study, from 1990 to 2021, the global incidence of AVC increased from 7.44 to 13.23 per 100,000, while its prevalence increased from 87.87 to 168.80 per 100,000, representing increases of 78% and 92%, respectively (1). Over the same period, the incidence and prevalence of AVC in China increased by 1.92-fold and 4.32-fold, respectively, exceeding the growth observed for many other cardiovascular diseases. As a chronic degenerative calcific disease, AVC may progress to aortic stenosis or insufficiency, impair ventricular outflow, and is an important cause of cardiovascular death in older adults (2, 3). The pathogenesis of AVC is complex and involves multiple processes, including inflammation, metabolic abnormalities, and mechanical stress. Established risk factors include hypertension, diabetes, obesity, and hyperlipidaemia (4–6). However, current prevention strategies targeting individual risk factors remain limited, and no specific pharmacological therapy or well-established preventive measure is currently available for AVC. Therefore, identifying comprehensive strategies associated with lower AVC burden remains an important clinical priority.

In 2022, the American Heart Association (AHA) introduced Life's Essential 8 (LE8), which provides a new framework for cardiovascular health assessment and disease prevention. LE8 includes four cardiovascular health behaviors (diet, physical activity, nicotine exposure, and sleep health) and four cardiovascular health factors (body mass index, non-high-density lipoprotein cholesterol, blood glucose, and blood pressure) (7, 8). The goal of LE8 is to optimize cardiovascular health and reduce the risk of cardiovascular diseases through the comprehensive management of these behaviors and factors. Although several prospective cohort studies have shown that higher LE8 scores are associated with lower risks of cardiovascular events, including coronary heart disease and stroke, the association between LE8 and AVC remains unclear (9–11). Therefore, based on the Chinese Kailuan cohort study (registration number: ChiCTR-TNC-11001489), we examined the association between long-term cumulative LE8 score and AVC and further evaluated the strength and heterogeneity of this association. Our findings may provide evidence to support cardiovascular health promotion and may have implications for early prevention strategies for AVC.

## 2 Subjects and methods

### 2.1 Study population

The Kailuan Study is a large prospective cohort based on the health management program for employees and retirees of the Kailuan Group in China. It was established to investigate the epidemiology of cardio-cerebrovascular diseases and their risk factors, as well as potential prevention and intervention strategies in this population (12, 13). Since 2006, participants have undergone standardized questionnaire assessments, clinical examinations, and laboratory tests every two years.

**Figure 1:**
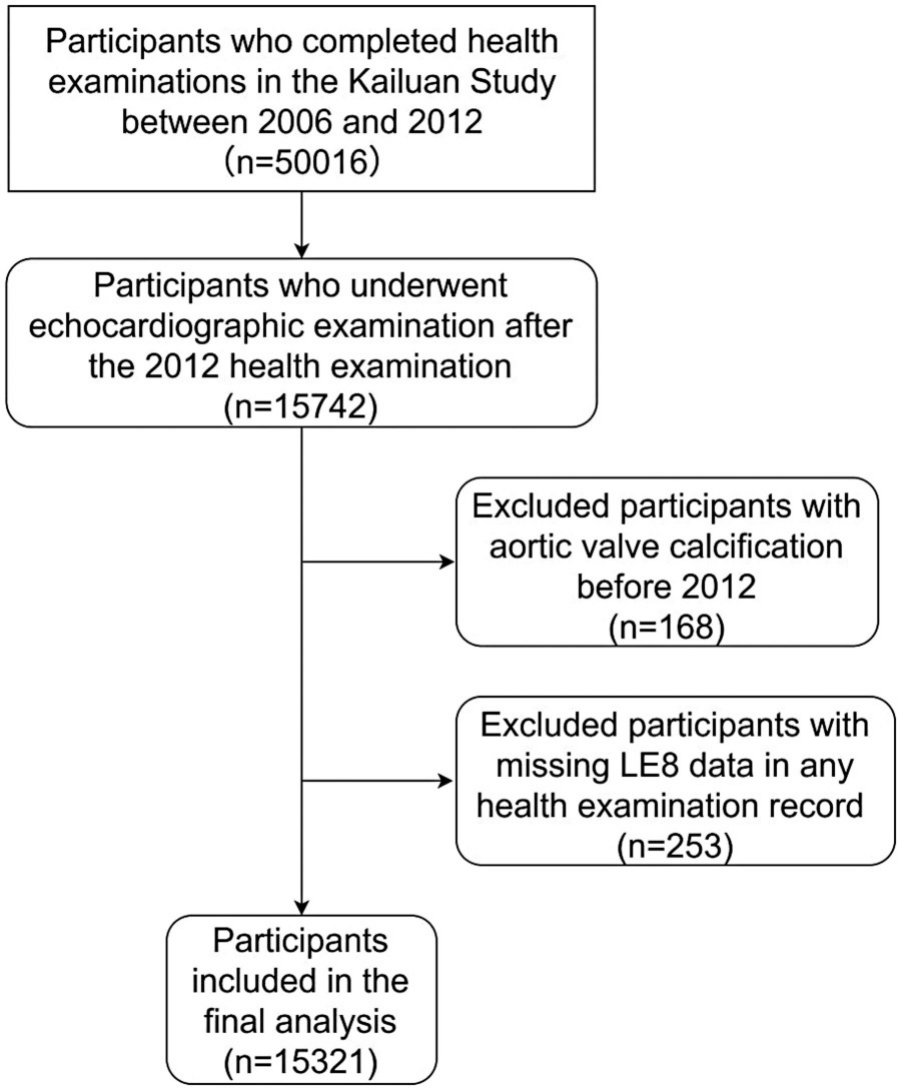
Flowchart of participant selection. The flowchart illustrates the process of participant inclusion and exclusion in the present study. A total of 50,016 participants who completed health examinations in the Kailuan Study between 2006 and 2012 were initially identified. Among them, 15,742 participants underwent echocardiographic examination after the 2012 health examination. We excluded 168 participants with prevalent aortic valve calcification before 2012 and 253 participants with missing LE8 data in any health examination record between 2006 and 2012. Finally, 15,321 participants were included in the analysis.

For the present analysis, we initially identified 50,016 participants who completed four consecutive health examinations between 2006 and 2012. Of these, 15,742 participants who underwent echocardiography after 2012 were considered potentially eligible. We excluded 168 participants with prevalent aortic valve calcification before 2012 and 253 participants with missing LE8 data in any examination between 2006 and 2012. After these exclusions, 15,321 participants were included in the final analysis (Figure 1). This study was conducted in accordance with the Declaration of Helsinki. Written informed consent was obtained from all participants, and the study protocol was approved by the Ethics Committee of Kailuan General Hospital (approval no. 2006-05).

### 2.2 Data collection methods

#### 2.2.1 General information collection

Basic information: Face-to-face interviews were conducted by uniformly trained medical staff using a standardized questionnaire. Information collected included demographic characteristics (sex, date of birth, education level, occupation type, and income level), lifestyle factors (sleep duration, physical activity time, smoking status, salt intake, tea consumption, and high-fat food intake), and health-related information (history of hypertension, diabetes, and hyperlipidaemia; use of related medications; and family history of myocardial infarction and stroke).

Anthropometric measurements: Height and weight were measured using an automated body measurement system while participants wore lightweight clothing and no shoes or headgear. Body mass index (BMI) was calculated as weight in kilograms divided by height in meters squared. Blood pressure was measured twice by trained nurses using calibrated mercury sphygmomanometers (electronic sphygmomanometers since 2014), with a 5 min interval after a 5 min rest in the seated position. If the difference between the two readings was ≥5 mmHg, a third measurement was obtained, and the average of the three readings was used for analysis.

Laboratory examination: On the morning of the physical examination day, 5 mL of fasting venous blood was collected by trained personnel. The following parameters were measured using a Hitachi 747 automatic analyzer (Hitachi 747, Hitachi, Tokyo, Japan): fasting blood glucose (FBG), total cholesterol (TC), high-density lipoprotein cholesterol (HDL-C), triglycerides (TG), serum creatinine (Cr), and high-sensitivity C-reactive protein (hs-CRP). Non-HDL cholesterol was calculated as total cholesterol minus HDL-C. The estimated glomerular filtration rate (eGFR) was calculated using the Chronic Kidney Disease Epidemiology Collaboration (CKD-EPI) equation. For men, if Cr ≤0.9 mg/dL, eGFR = 141 × (Cr/0.9)^−0.411^ × (0.993) ^age^; if Cr > 0.9 mg/dL, the eGFR = 141 × (Cr/0.9)^−1.209^ × (0.993) ^age^. For females, if Cr ≤ 0.7 mg/dL, the eGFR = 144 × (Cr/0.7)^−0.329^ × (0.993) ^age^; if Cr > 0.7 mg/dL, the eGFR = 144 × (Cr/0.7)^−1.209^ × (0.993) ^age^.

#### 2.2.2 Calculation of TWC-LE8 scores

In this study, time-weighted cumulative Life's Essential 8 (TWC-LE8) scores were derived from data collected during routine health examinations. In accordance with the AHA guidelines and taking into account characteristics of the Chinese population, the scoring criteria for diet and BMI were modified to construct a Kailuan-adapted LE8 scoring system, which has been widely used in related studies (14, 15). Previous studies have shown that cardiovascular health in the Chinese population is closely associated with salt intake, high-fat food consumption, and tea-drinking habits (16, 17). Therefore, in the Kailuan Study, the unweighted mean scores for salt intake, tea consumption frequency, and high-fat food intake were used to replace the AHA Dietary Approaches to Stop Hypertension (DASH)-style diet score. In addition, BMI scoring was modified according to the Chinese criteria for overweight and obesity as follows: BMI < 23.0 kg/m^2^: 100 points; BMI 23.0–24.9 kg/m^2^: 75 points; BMI 25.0–29.9 kg/m^2^: 50 points; BMI 30.0–34.9 kg/m^2^: 25 points; and BMI > 35.0 kg/m^2^: 0 points (18). Detailed scoring rules for all components are provided in Supplementary Table S1. Each LE8 component was scored from 0 to 100, and the total LE8 score was calculated as the unweighted mean of the eight component scores, with higher scores indicating better cardiovascular health.

TWC-LE8 scores were calculated using LE8 assessments from four health examinations conducted in 2006, 2008, 2010, and 2012. The formula was as follows:

$$\begin{array}{rl} \mathrm{TWC}\text{-}\mathrm{LE}8 & =[((\mathrm{LE}8_{2006}+\mathrm{LE}8_{2008})/2)\times (t_{2008}-t_{2006})\\ & +((\mathrm{LE}8_{2008}+\mathrm{LE}8_{2010})/2)\\ & \times (t_{2010}-t_{2008})\\ & +((\mathrm{LE}8_{2010}+\mathrm{LE}8_{2012})/2)\\ & \times (t_{2012}-t_{2010})]/(t_{2012}-t_{2006}). \end{array}$$

LE8_2006_, LE8_2008_, LE8_2010_, and LE8_2012_ were the LE8 scores in 2006, 2008, 2010, and 2012, respectively. The time intervals between consecutive LE8 measurements were as follows: *t*_2008_−*t*_2006_, *t*_2010_−*t*_2008_, and *t*_2012_−*t*_2010_, with *t*_2012_−*t*_2006_ representing the total time interval from the first to the last LE8 measurement. On the basis of recommendations from the AHA writing group, the TWC-LE8 scores were categorized into three cardiovascular health (CVH) levels: Low CVH: TWC-LE8 < 50; Moderate CVH: 50 ≤ TWC-LE8 < 80; High CVH: TWC-LE8 ≥ 80 (9). These categories reflect the overall cardiovascular health status, with higher scores indicating better CVH.

#### 2.2.3 Determination of AVC

Echocardiographic examinations were performed by sonographers who had received standardized training and had more than 5 years of clinical experience, in accordance with relevant practice guidelines (19). Participants were examined in the left lateral decubitus position using PHILIPS IE Elite, IE33, and EPIQ 7C color Doppler echocardiography systems equipped with an S5-1 phased-array probe (frequency, 1–5 MHz). The echogenic characteristics and mobility of the aortic valve were assessed under standard parasternal long-axis and short-axis views. AVC was classified as either present or absent. A positive diagnosis of AVC was made if either of the following criteria was met: (1) thickening of the aortic valve leaflets (thickness≥1 mm), increased echogenicity, leaflet stiffness, and restricted motion; or (2) localized plaque-like echo enhancement of the aortic annulus greater than or equal to that of the aortic root. All echocardiographic images were independently evaluated in a blinded manner by two senior sonographers with at least 5 years of experience in valvular disease, according to guideline criteria (20). In cases of disagreement, a third expert was consulted, and the final diagnosis was determined by consensus. In addition, a random sample of echocardiographic images was reviewed by another senior echocardiography expert to ensure the reliability of AVC diagnosis.

### 2.3 Statistical methods

All statistical analyses were performed using SAS version 9.4. Normally distributed continuous variables are presented as mean ± standard deviation (SD), and comparisons between groups were conducted using analysis of variance. Non-normally distributed continuous variables are presented as median (P25-P75), and comparisons between groups were performed using the rank-sum test. Categorical variables are presented as frequencies and percentages, and comparisons between groups were conducted using the chi-square test.

Multivariable logistic regression was used to examine the association between TWC-LE8 score groups and AVC. Two models were constructed. Model 1 was adjusted for age and sex. Model 2 was further adjusted for alcohol consumption, education level, average income, occupation, family history of myocardial infarction, family history of stroke, eGFR, and hs-CRP. To assess the robustness of our findings, we performed sensitivity analyses by additionally adjusting for LE8 scores at the first and last health examinations based on Model 2, and by further excluding participants with atherosclerotic cardiovascular disease (ASCVD) and those using lipid-lowering medications. To evaluate the contribution of each TWC-LE8 component to the association between TWC-LE8 score and AVC, multivariable logistic regression analyses were repeated after sequentially excluding each component and recalculating the time-weighted cumulative mean of the remaining seven components. To further examine whether the association between TWC-LE8 score and AVC differed by sex (male/female) and age (<55/≥55 years), stratified analyses were performed, and interaction terms between sex, age, and TWC-LE8 score were introduced into the multivariable logistic regression models. All models were adjusted for the aforementioned covariates. To assess the dose-response relationship between TWC-LE8 score and AVC, restricted cubic spline (RCS) regression models were fitted with three knots placed at the 10th, 50th, and 90th percentiles according to the Akaike information criterion (AIC). The likelihood ratio test was used to assess deviation from linearity. All statistical tests were two-sided, and *P* < 0.05 was considered statistically significant.

## 3 Results

### 3.1 Baseline characteristics of the study population

A total of 15,321 participants were included in this study, with a mean age of 63.79 ± 10.74 years, and 76.44% were men. According to CVH level, 1,913 (12.5%), 13,021 (85.0%), and 387 (2.5%) participants were categorized into the low, moderate, and high CVH groups, respectively. With increasing TWC-LE8 scores, the proportions of women, participants with a higher education level (college or above), never drinkers, and other blue-collar workers increased progressively. Compared with the low CVH group, the high CVH group was younger and had a lower prevalence of family history of cardiovascular disease (myocardial infarction and stroke), lower inflammatory levels, and better renal function (all *P* < 0.001). Detailed baseline characteristics are shown in Table 1.

**Table 1:** Baseline characteristics of participants stratified by TWC-LE8 score groups.

	Total	Low CVH	Moderate CVH	High CVH	P-value
	(*N* = 15,321)	(*N* = 1,913)	(*N* = 13,021)	(*N* = 387)	
Age, years	63.79 ± 10.74	62.38 ± 9.30	64.22 ± 10.79	56.24 ± 12.16	<0.001
Women, %	3610 (23.56)	147 (7.68)	3179 (24.41)	284 (73.39)	<0.001
Men, %	11711 (76.44)	1766 (92.32)	9842 (75.59)	103 (26.61)	<0.001
Education level					
Illiterate	109 (0.71)	14 (0.73)	95 (0.73)	NA	<0.001
Primary	1351 (8.82)	202 (10.56)	1138 (8.74)	11 (2.84)	<0.001
Middle school	10899 (71.14)	1355 (70.83)	9329 (71.65)	215 (55.56)	<0.001
High school	2059 (13.44)	258 (13.49)	1722 (13.22)	79 (20.41)	<0.001
University or above	903 (5.89)	84 (4.39)	737 (5.66)	82 (21.19)	<0.001
Alcohol consumption					
Never	8860 (57.83)	670 (35.02)	7877 (60.49)	313 (80.88)	<0.001
Past	572 (3.73)	98 (5.12)	471 (3.62)	3 (0.78)	<0.001
Light	2759 (18.01)	423 (22.11)	2280 (17.51)	56 (14.47)	<0.001
Heavy	3130 (20.43)	722 (37.74)	2393 (18.38)	15 (3.88)	<0.001
Income level					
Missing	515 (3.36)	64 (3.35)	446 (3.43)	5 (1.29)	<0.001
<1000¥ per mo	4283 (28.93)	658 (35.59)	3533 (28.10)	92 (24.08)	<0.001
1000-3000¥ per mo	8368 (56.52)	877 (47.43)	7260 (57.73)	231 (60.47)	<0.001
3000-5000¥ per mo	1188 (8.02)	178 (9.63)	988 (7.86)	22 (5.76)	<0.001
>5000¥ per mo	967 (6.53)	136 (7.36)	794 (6.31)	37 (9.69)	<0.001
Occupation					
Missing	4 (0.03)	1 (0.05)	3 (0.02)	NA	<0.001
Coalminers	4588 (29.95)	809 (42.29)	3753 (28.82)	26 (6.72)	<0.001
Other blue collars	10667 (69.62)	1091 (57.03)	9217 (70.79)	359 (92.76)	<0.001
White collars	62 (0.40)	12 (0.63)	48 (0.37)	2 (0.52)	<0.001
Family history of myocardial infarction	708 (4.62)	137 (7.16)	556 (4.27)	15 (3.88)	<0.001
Family history of stroke	1894 (12.36)	320 (16.73)	1533 (11.77)	41 (10.59)	<0.001
eGFR, mL/min/1.73 m^2^	86.78 ± 13.85	90.46 ± 13.68	86.06 ± 13.73	92.62 ± 14.27	<0.001
hs-CRP, mg/L	0.23 ± 0.38	0.35 ± 0.34	0.22 ± 0.38	0.02 ± 0.38	<0.001
Missing	4 (0.03)	1 (0.05)	3(0.02)	NA	<0.001
TWC-LE8	61.30 ± 9.76	44.92 ± 4.29	63.08 ± 7.29	82.72 ± 2.31	<0.001

eGFR, estimated glomerular filtration rate; hs-CRP, high-sensitivity C-reactive protein; TWC-LE8, time-weighted cumulative life's essential 8 scores; CVH, cardiovascular health; AVC, aortic valve calcification.

Normally distributed data were reported as mean ± standard deviation. Non-normally distributed data were reported as median.

### 3.2 Association between CVH and AVC

A total of 2,894 participants were identified as having AVC in this study. The prevalence of AVC was 25.88%, 18.26%, and 5.43% in the low, moderate, and high CVH groups, respectively. Multivariable logistic regression was performed to examine the association between TWC-LE8 score and AVC. In Model 1, adjustments were made for age and sex. Model 2 was further adjusted for alcohol consumption, education level, average income, occupation, family history of myocardial infarction, family history of stroke, eGFR, and hs-CRP. Compared with the low CVH group, the moderate CVH group was associated with 47% lower odds of AVC (OR = 0.53, 95% CI: 0.47–0.61), whereas the high CVH group was associated with 75% lower odds of AVC (OR = 0.25, 95% CI: 0.15–0.40). In addition, each 10-point increase in CVH score was associated with 34% lower odds of AVC (OR = 0.66, 95% CI: 0.62–0.69). Detailed results are presented in Table 2.

**Table 2:** Detection rate of AVC event and multivariable logistic regression analysis results by different CVH groups.

Group	Cases/Total	AVC prevalence	Model 1	Model 2
		(%)	OR (95% CI)	OR (95% CI)
Low CVH	495/1913	25.88	Ref.	Ref.
Moderate CVH	2378/13021	18.26	0.53 (0.47, 0.59)	0.53 (0.47, 0.61)
High CVH	21/387	5.43	0.23 (0.15, 0.37)	0.25 (0.15, 0.40)
CVH per 10 points increment			0.66 (0.63, 0.70)	0.66 (0.62, 0.69)
P-value			<0.001	<0.001

**Model 1:** Age and sex.

**Model 2:** Model 1 + alcohol consumption history, education level, average income, occupation, family history of myocardial infarction, family history of stroke, eGFR and hs-CRP.

eGFR, estimated glomerular filtration rate; hs-CRP, high-sensitivity C-reactive protein; CVH, cardiovascular health; AVC, aortic valve calcification.

### 3.3 Sensitivity analysis

**Figure 2:**
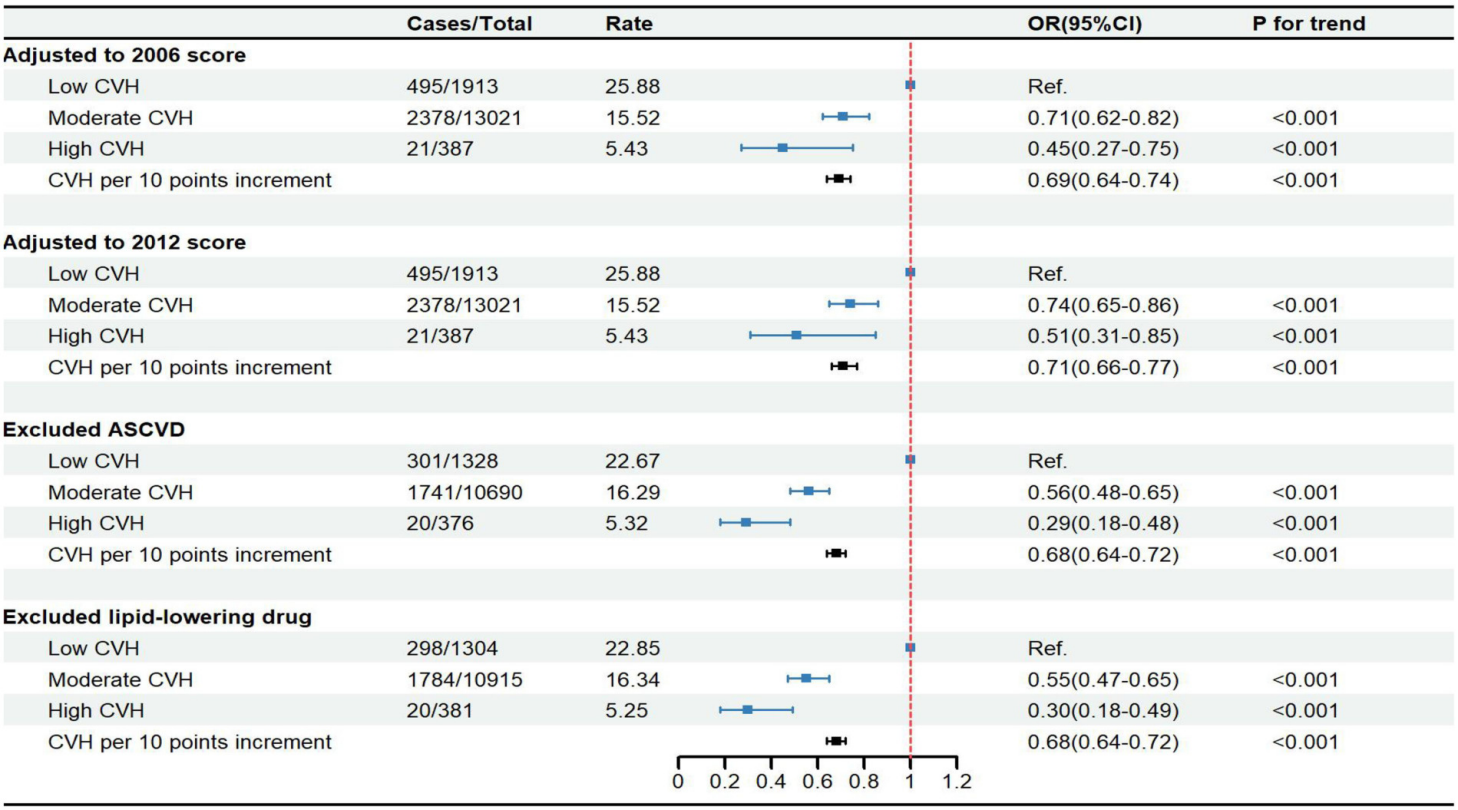
Forest plot of sensitivity analyses for the association between time-weighted cumulative Life's Essential 8 (TWC-LE8) and aortic valve calcification (AVC). The figure displays odds ratios (95% confidence intervals) derived from multiple sensitivity analyses. The number of cases, total participants, and AVC prevalence (%) are presented for each subgroup. **Note:** Models were adjusted for age, sex, alcohol consumption history, education level, average income, occupation, family history of myocardial infarction, family history of stroke, eGFR and hs-CRP. **Abbreviations:** eGFR, estimated glomerular filtration rate; hs-CRP, high-sensitivity C-reactive protein; CVH, cardiovascular health; AVC, aortic valve calcification.

To assess the robustness of our findings, we conducted several sensitivity analyses to further examine the association between TWC-LE8 score and AVC. Based on Model 2, we additionally adjusted for the LE8 score at the first and last health examinations. Compared with the low CVH group, the high CVH group was associated with 55% lower odds of AVC after further adjustment for the LE8 score at the first health examination (OR = 0.45, 95% CI: 0.27–0.75) and with 49% lower odds of AVC after further adjustment for the LE8 score at the last health examination (OR = 0.51, 95% CI: 0.31–0.85). In addition, each 10-point increase in CVH score was associated with 31% lower odds of AVC in one analysis (OR = 0.69, 95% CI: 0.64–0.74) and 29% lower odds in the other (OR = 0.71, 95% CI: 0.66–0.77). To minimize the potential influence of ASCVD, we repeated the analysis after excluding participants with ASCVD. In this subset, the high CVH group was associated with 71% lower odds of AVC (OR = 0.29, 95% CI: 0.18–0.48), and each 10-point increase in CVH score was associated with 32% lower odds of AVC (OR = 0.68, 95% CI: 0.64–0.72). After excluding participants using lipid-lowering medications, the high CVH group was associated with 70% lower odds of AVC (OR = 0.30, 95% CI: 0.18–0.49), and each 10-point increase in CVH score was associated with 32% lower odds of AVC (OR = 0.68, 95% CI: 0.64–0.72). Detailed results are presented in Figure 2.

### 3.4 Stratified analysis

**Figure 3:**
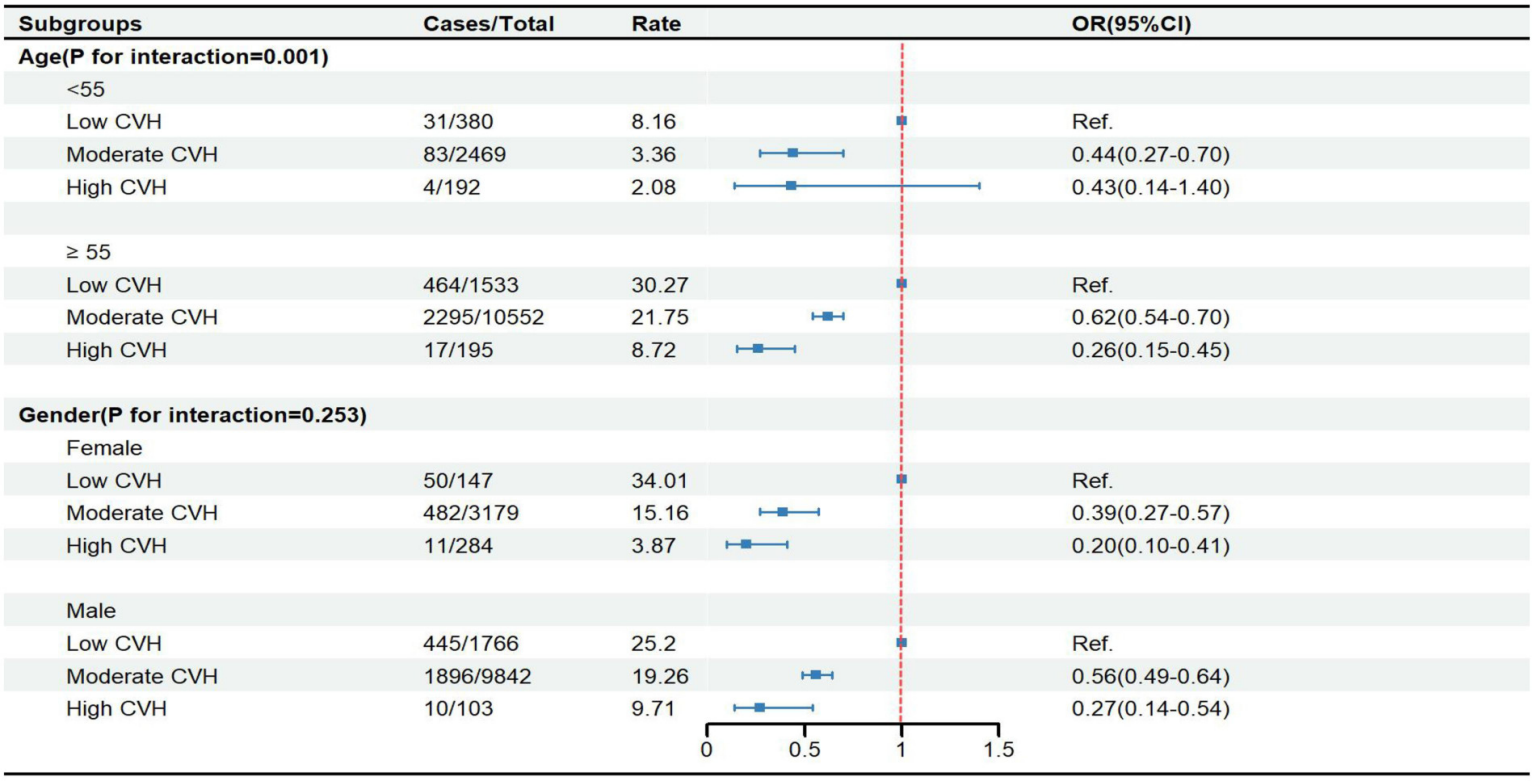
Association between cardiovascular health (CVH) and aortic valve calcification (AVC) stratified by sex and age. Forest plots show the odds ratios (95% confidence intervals) for the association between CVH levels and AVC in subgroups defined by sex and age. **Note:** Models were adjusted for age, sex, alcohol consumption history, education level, average income, occupation, family history of myocardial infarction, family history of stroke, eGFR and hs-CRP. **Abbreviations:** eGFR, estimated glomerular filtration rate; hs-CRP; CVH, cardiovascular health; AVC, aortic valve calcification.

Stratified analyses were conducted according to sex and age. No significant interaction was observed between sex and the association between TWC-LE8 score and AVC (P for interaction = 0.253), whereas a significant interaction was observed for age (P for interaction = 0.01). Compared with the low CVH group, the high CVH group was associated with 80% lower odds of AVC in women (OR = 0.20, 95% CI: 0.10–0.41) and 73% lower odds of AVC in men (OR = 0.27, 95% CI: 0.14–0.54). In the age-stratified analyses, the association between high CVH and AVC did not reach statistical significance among participants aged <55 years. However, among those aged ≥55 years, the high CVH group was associated with 74% lower odds of AVC (OR = 0.26, 95% CI: 0.15–0.45). Detailed results are presented in Figure 3.

### 3.5 Relative contribution of individual LE8 components to the association between CVH and AVC

**Figure 4:**
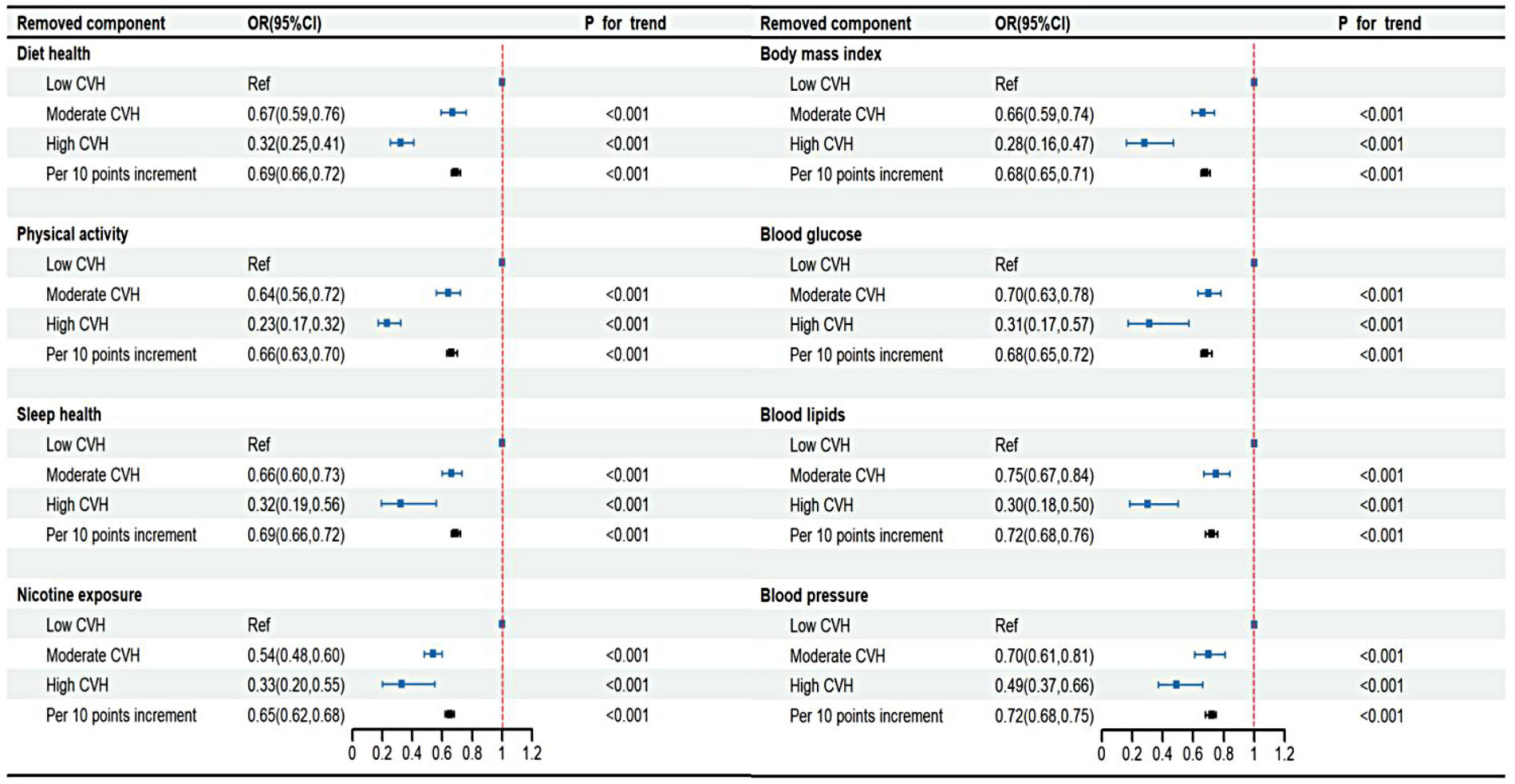
Logistic regression analysis of the association between cardiovascular health (CVH) and aortic valve calcification (AVC) after excluding individual LE8 components one by one. Forest plots present odds ratios (95% confidence intervals) based on time-weighted cumulative mean CVH scores derived from the remaining seven components. **Note:** To analyze the dose–response relationship between TWC-LE8 scores and AVC, we selected nodes at the 10th, 50th, and 90th percentiles of the TWC-LE8 score. Multivariable regression models were employed, adjusting for potential confounders, including age, sex, alcohol consumption history, education level, average income, occupation, family history of myocardial infarction, family history of stroke, eGFR and hs-CRP. **Abbreviations:** eGFR, estimated glomerular filtration rate; hs-CRP; TWC-LE8, time-weighted cumulative life's essential 8 scores; AVC, aortic valve calcification.

We repeated the logistic regression analyses by sequentially excluding each LE8 component and recalculating the LE8 score based on the remaining seven components. The results indicated that blood pressure had a relatively greater contribution to the association between CVH and AVC, whereas physical activity had a relatively smaller contribution. Detailed results are shown in Figure 4.

### 3.6 Restricted cubic spline analysis

**Figure 5:**
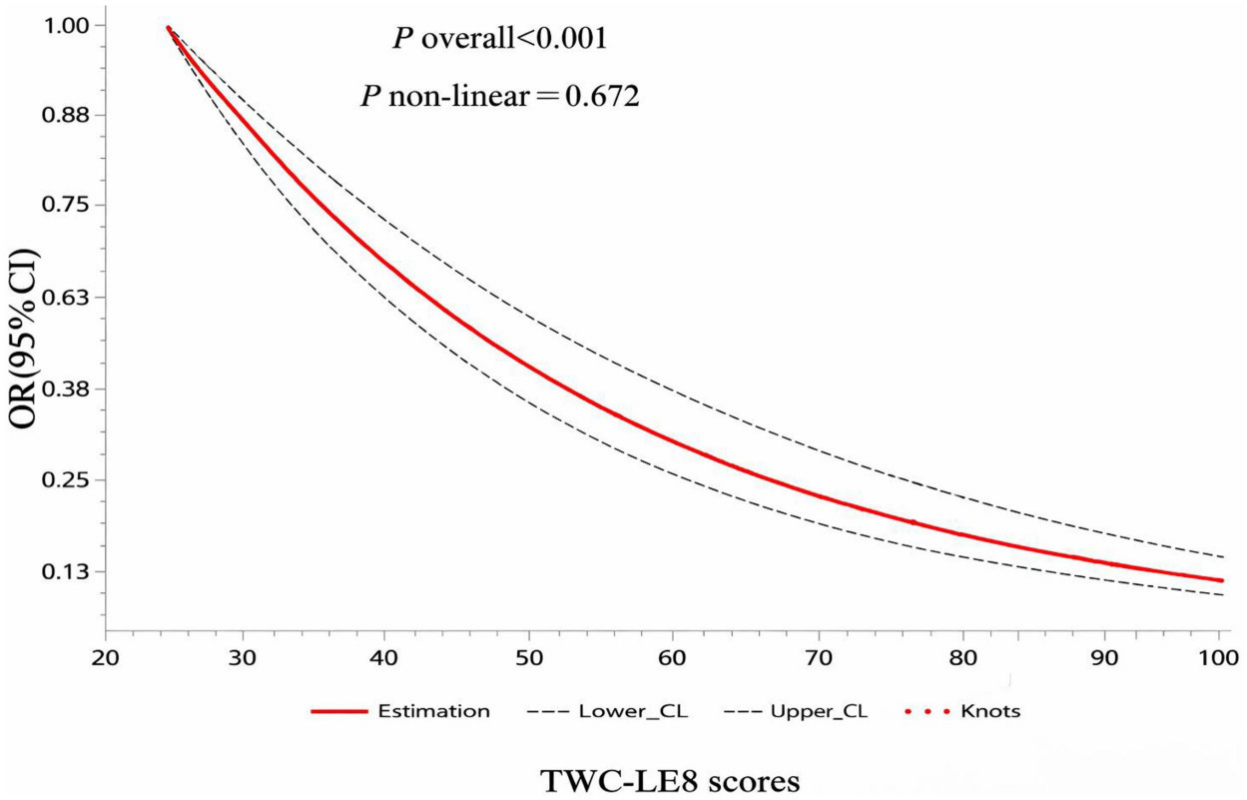
Restricted cubic spline analysis of the association between time-weighted cumulative Life's Essential 8 (TWC-LE8) scores and aortic valve calcification (AVC). The restricted cubic spline plot illustrates the dose–response relationship between TWC-LE8 scores and the risk of AVC. Knots were placed at the 10th, 50th, and 90th percentiles of the TWC-LE8 score distribution. Multivariable logistic regression models were used with adjustment for age, sex, alcohol consumption, education level, average income, occupation, family history of myocardial infarction, family history of stroke, estimated glomerular filtration rate (eGFR), and high-sensitivity C-reactive protein (hs-CRP). The solid red line represents the estimated odds ratio, and the dashed lines indicate the 95% confidence intervals.

Restricted cubic spline analysis showed a significant dose-response association between TWC-LE8 score and AVC, with no evidence of nonlinearity (P overall < 0.001, P for nonlinearity = 0.672). Higher TWC-LE8 score was associated with progressively lower odds of AVC. Detailed results are shown in Figure 5.

## 4 Discussion

To our knowledge, this study is the first to systematically examine the association between TWC-LE8 score and AVC based on large-scale population data. We observed a significant inverse association between TWC-LE8 score and AVC, with a dose-response pattern that appeared to vary by age. In addition, among the individual components of TWC-LE8, blood pressure showed the greatest contribution to the association with AVC.

Previous studies have shown that higher LE8 scores are significantly associated with lower risks of cardiovascular diseases, with evidence of dose-response relationships. Higher CVH levels have been associated with 44%, 67%, and 68% lower risks of coronary heart disease, stroke, and heart failure, respectively, and each 10-point increase in LE8 score has been associated with a 17% lower risk of cardiovascular disease (11, 21–23). Similarly, a meta-analysis reported that high CVH status was associated with a 13% to 81% lower risk of cardiovascular disease (24). Building on these findings, our study extends the investigation of LE8 to AVC and showed that higher TWC-LE8 scores were also significantly associated with lower odds of AVC. Compared with the low CVH group, the high CVH group was associated with 75% lower odds of AVC, and each 10-point increase in CVH score was associated with 34% lower odds of AVC. Notably, most previous studies have relied on a single assessment of CVH status, and only a few have evaluated the association of long-term LE8 trajectories with outcomes such as stroke and heart failure (14, 15). However, evidence regarding the association between long-term CVH changes and other cardiovascular outcomes, particularly valvular diseases, remains limited. To our knowledge, this study is the first to systematically examine the association between 8-year TWC-LE8 score and AVC. Even after additional adjustment for LE8 scores at the initial and final health examinations, the association remained robust. These findings suggest that long-term CVH status may be relevant to AVC and add to the growing body of evidence supporting the potential value of LE8 in valvular disease prevention.

Our study showed a significant interaction between age and the association of TWC-LE8 score with AVC (P for interaction < 0.05). Age-stratified analyses showed that, among individuals aged 55 years and older, the high CVH group was associated with 74% lower odds of AVC (OR = 0.26, 95% CI: 0.15–0.45), whereas among those younger than 55 years, the association was not statistically significant (OR = 0.43, 95% CI: 0.14–1.40). This age-related difference may be partly explained by the greater cumulative exposure to cardiovascular risk factors and the progressive accumulation of cardiovascular damage with aging. In older adults, the cardiovascular system may be more vulnerable to adverse health states and therefore more likely to reflect differences in overall cardiovascular health (25, 26). In addition, the relatively low prevalence of AVC among younger individuals may have limited the statistical power to detect a significant association in this subgroup (27, 28). In our study, the prevalence of AVC in the high CVH group was 2.08% among participants younger than 55 years and 8.72% among those aged 55 years and older, which may have influenced the observed statistical significance. Although no significant interaction between sex and TWC-LE8 score was observed in relation to AVC (P for interaction = 0.253), stratified analyses showed a somewhat stronger inverse association in women than in men, with 80% and 73% lower odds of AVC, respectively, in the high CVH group. A large cohort study based on the UK Biobank similarly reported that the association of LE8 with lower risks of cardiovascular diseases, including coronary heart disease and stroke, was stronger in women than in men (23). This difference may be related to the anti-inflammatory and vasculoprotective effects of estrogen. Previous studies have shown that estrogen may reduce the osteogenic differentiation of valvular interstitial cells by inhibiting the nuclear factor-*κ*B pathway, thereby delaying the calcification process (29). In addition, greater adherence to healthy behaviors among women, such as smoking cessation and dietary control, may further enhance the cumulative benefits of CVH (30).

Although the mechanisms underlying the association between LE8 and AVC are not fully understood, several biological pathways may be involved. First, clinical studies have shown that blood pressure control is closely related to slower AVC progression (31). In our leave-one-out analysis, exclusion of the blood pressure component resulted in a relatively greater attenuation of the association between LE8 and AVC, suggesting that blood pressure may make an important contribution to the overall association between LE8 and AVC. Long-term blood pressure control may help reduce mechanical stress on the aortic valve, inhibit angiotensin II-mediated inflammatory responses, and delay the osteogenic differentiation of valvular interstitial cells, which may in turn be associated with less calcification (32). Second, lipid control, such as lowering LDL-C, may reduce oxidized LDL deposition in the valve and thereby attenuate local inflammation and calcification (33). In addition, a healthy diet and blood glucose control may reduce valve collagen cross-linking and calcium salt deposition by inhibiting the accumulation of advanced glycation end products (AGEs) and blocking activation of the receptor for AGEs (RAGE) signaling pathway (34). Although physical activity showed a relatively smaller contribution in this study, it may still be indirectly associated with valvular protection through improvements in systemic metabolism, such as increased adiponectin levels and reduced systemic inflammation (35). Moreover, lower tobacco exposure, healthy sleep patterns, and optimal body weight may collectively reduce oxidative stress, inflammation, and metabolic disturbances, thereby benefiting overall cardiovascular health. Taken together, these findings suggest that the integrated healthy behaviors and factors represented by LE8 may be associated with lower odds of AVC through pathways related to mechanical stress, inflammation, oxidative stress, and metabolic dysregulation.

## 5 Study strengths and limitations

This study has several strengths. First, the 8-year time-weighted cumulative LE8 score was used to more comprehensively reflect long-term cardiovascular health status, reduce potential short-term fluctuations associated with a single measurement, and improve the stability and reliability of the findings. Second, the data were derived from real-world clinical practice, which enhances the external validity of the study and may provide useful evidence for clinical decision-making and public health policy. Third, the LE8 scoring system used in this study was adapted to better reflect the health characteristics and lifestyle patterns of Chinese and other Asian populations, thereby making the findings more population-specific and providing a more relevant basis for cardiovascular health assessment in these groups.

Despite these strengths, several limitations should be acknowledged. First, because this was an observational study, residual confounding could not be completely excluded, and causal inference remains limited. Although multiple potential confounders were adjusted for, unmeasured factors, including genetic susceptibility, environmental exposures, and detailed dietary patterns, may still have influenced the results. Future studies incorporating more detailed lifestyle measurements and stronger causal designs, such as Mendelian randomization analyses, are needed to further validate our findings. Second, AVC in this study was assessed primarily by echocardiography rather than computed tomography (CT). Although echocardiography is widely used and feasible in large-scale studies, it is less sensitive for detecting mild or early calcification. Therefore, some mild AVC cases may have been missed, possibly resulting in an underestimation of AVC prevalence and attenuation of the observed association between LE8 scores and AVC (36). Third, the study population consisted predominantly of men and occupational participants from a Chinese industrial community, which may limit the generalizability of the findings to women, non-occupational populations, and other ethnic groups. Further studies in more diverse populations are needed to confirm the external validity of our findings. Finally, although the 8-year cumulative LE8 score used as the exposure variable may better reflect long-term health status, the potential influence of year-to-year variation and temporal trends in LE8 score on AVC was not further examined. Future studies should incorporate changes in LE8 score across different time windows to explore the association between dynamic patterns and AVC.

To our knowledge, this is the first study to use TWC-LE8 score to examine its association with AVC. By integrating multiple lifestyle and health factors over a prolonged period, this approach provides a more comprehensive framework for cardiovascular risk assessment. Compared with traditional analyses based on a single time point or individual factors, our study captured the cumulative long-term pattern of eight cardiovascular health indicators. This design may provide a useful perspective for future research on cardiovascular health and valvular disease prevention.

## Data Availability

The data analyzed in this study is subject to the following licenses/restrictions: The datasets analyzed during the current study are securely stored at Kailuan Hospital and have been fully de-identified. Data are available from the corresponding author upon reasonable request and subject to institutional and publisher data-sharing policies. Requests to access these datasets should be directed to Shouling Wu, drwusl@163.com.
